# Maize *male sterile 33* encodes a putative glycerol-3-phosphate acyltransferase that mediates anther cuticle formation and microspore development

**DOI:** 10.1186/s12870-018-1543-7

**Published:** 2018-12-03

**Authors:** Lei Zhang, Hongbing Luo, Yue Zhao, Xiaoyang Chen, Yumin Huang, Shuangshuang Yan, Suxing Li, Meishan Liu, Wei Huang, Xiaolan Zhang, Weiwei Jin

**Affiliations:** 1 National Maize Improvement Center of China, Beijing Key Laboratory of Crop Genetic Improvement, Key Laboratory of Crop Heterosis and Utilization, Ministry of Education (MOE), Center for Crop Functional Genomics and Molecular Breeding, China Agricultural University, Beijing, 100193 China; 2 College of Agronomy, Southern Regional Collaborative Innovation Center for Grain and Oil Crops, Hunan Agricultural University, Changsha, 410128 China; 3 Department of Vegetable Sciences, Beijing Key Laboratory of Growth and Developmental Regulation for Protected Vegetable Crops, China Agricultural University, Beijing, 100193 China

**Keywords:** Maize, Male sterile, MS33, Anther cuticle, Tapetum, GPAT

## Abstract

**Background:**

The anther cuticle, which is primarily composed of lipid polymers, is crucial for pollen development and plays important roles in sexual reproduction in higher plants. However, the mechanism underlying the biosynthesis of lipid polymers in maize (*Zea mays.* L.) remains unclear.

**Results:**

Here, we report that the maize male-sterile mutant *shrinking anther 1* (*sa1*), which is allelic to the classic mutant *male sterile 33* (*ms33*)*,* displays defective anther cuticle development and premature microspore degradation. We isolated *MS33* via map-based cloning. *MS33* encodes a putative glycerol-3-phosphate acyltransferase and is preferentially expressed in tapetal cells during anther development. Gas chromatography-mass spectrometry revealed a substantial reduction in wax and cutin in *ms33* anthers compared to wild type. Accordingly, RNA-sequencing analysis showed that many genes involved in wax and cutin biosynthesis are differentially expressed in *ms33* compared to wild type.

**Conclusions:**

Our findings suggest that *MS33* may contribute to anther cuticle and microspore development by affecting lipid polyester biosynthesis in maize.

**Electronic supplementary material:**

The online version of this article (10.1186/s12870-018-1543-7) contains supplementary material, which is available to authorized users.

## Background

The pollen grains of angiosperms are produced in the anther compartment of the flower stamen. In maize, each male floret has three anthers, each with four lobes. These four lobes have similar structures and are attached to a central core connected to the vascular tissue. After morphogenesis, each anther differentiates into a four-layered structure. From the exterior to the interior, the centrally located microspores are covered by the epidermis, endothecium, middle layer, and tapetum [1, 2].

Male gametophyte development requires the functional cooperation of gametophytic and sporophytic tissues. The tapetum contributes to microspore development by providing energy and structural materials [3–5]. Mature pollen is covered by a complex exine, which is highly resistant to physical and chemical degradation and thus protects male gametophytes against drought, irradiation, and other environmental stresses [6–8]. The main component of the pollen exine is sporopollenin, a biopolymer formed by lipid monomers covalently coupled by ether and ester linkages. The major lipid precursors of sporopollenin include straight-chain fatty acids and oxygenated aromatic monomers, such as p-coumaric (C9) and ferulic (C10) acids, all of which are synthesized in the tapetum. After meiosis in the anther is complete, sporopollenin is secreted by the tapetum, transported to the microspore surface, and used for pollen exine formation [9, 10].

The epidermis of the anther is covered by the cuticle, a chemically stable layer that protects anthers from dehydration during development [8, 11]. Like the surface layers of vegetative organs, such as leaves and stems, the anther cuticle comprises two types of lipophilic biopolymers, cutin and wax. Cutin is composed of hydroxylated and epoxy C16 and C18 fatty acids, and wax is mainly formed by long-chain fatty acids [6, 9, 10, 12, 13]. The biosynthetic pathway of cutin has been elucidated gradually in resent researches, which consist of monomer synthesis, export and polymerization [14, 15]. The cutin monomers are synthesized from the plastid-derived 16C and 18C fatty acids in the endoplasmic reticulum, with the esterification of long-chain acyl-CoA synthetases (LACS) and the oxidation of cytochrome P450 oxidases from the CYP86 and CYP77 subfamilies [15, 16]. Then, the mature monoacylglycerol cutin monomers are generated by transfer of the acyl group from acyl-CoA to glycerol-3-phosphate under the catalysis of GPAT. The ABC transporters export the cutin monomers from endoplasmic reticulum to the site of polymerization [15]. Wax biosynthesis begins with a de novo C16 or C18 fatty acid in the plastid. Then, the LACS catalyze the long-chain fatty acid compounds to C16 or C18 acyl-CoA and then transferred to the endoplasmic reticulum. The fatty acid elongase (FAE) complex catalyze the C16 or C18 acyl-CoA to very-long-chain fatty acids (VLCFAs) with predominant chain lengths ranging from 24 to 36 carbons through several cycles of reaction. The aliphatic wax constituents are generated from VLCFAs by the alcohol-forming pathways, which give rise to primary alcohols and wax eaters, and the alkane-forming pathways, which produce aldehydes, alkanes, secondary alcohols, and ketones [17, 18].

In recent years, many genes have been identified to contribute to anther cuticle and pollen exine development, such as *AMS1* (*ABORTED MICROSPORES*) [7], *MS1* (*MALE STERILITY1*) [19], *MS2* (*MALE STERILITY2*) [20], *FLP1* (*FACELESS POLLEN1*) [21], *DEX1* (*DEFECTIVE IN EXINE PATTERN FORMATION1*) [22], *NEF1* (*NO EXINE FORMATION1*) [23], *CYP703A2* [24], *ACOS5* (*ACYL-COA SYNTHETASE5*) [25], and *CYP704B1* [26] in *Arabidopsis thaliana*, as well as the rice genes *TDR* (*TAPETUM DEGENERATION RETARDATION*) [27], *GAMYB* [28], *PDA1* (*POST-MEIOTIC DEFICIENT ANTHER1*) [29], *PTC1* (*PERSISTENT TAPETAL CELL1*) [30], *OsC6* [31], *WDA1* (*WAX-DEFICIENT ANTHER1*) [32], *CYP703A3* [10], *CYP704B2* [33], and *NP1* (*NO POLLEN1*) [34]. These genes are involved in fatty acid biosynthesis, modification, transport, and metabolism. Mutations in these genes result in abortion of the male gamete. However, the role of fatty acids in polyester formation is currently unclear [7, 9, 10].

In *Arabidopsis*, several glycerol-3-phosphate acyltransferase (GPAT) family members have been shown to participate in epidermal polyester formation in vegetative tissues [8]. GPAT catalyzes the esterification of a fatty acyl from acyl-CoA to the sn-2 position of glycerol-3-phosphate (G3P), producing lysophosphatidate (LPA). LPA is a substrate for lipid monomer biosynthesis during cutin and wax production in plants [35]. Genetic analysis suggested that GPAT is involved in pollen development in *Arabidopsis*. For example, the *gpat1* mutant exhibits anomalous pollen coat structure and very poor male fertility, and the *gpat1 gpat6* double mutant is completely male sterile [36]. One GPAT family member, OsGPAT3, plays a critical role in anther cuticle and male gamete development in rice [37]. However, whether GPATs directly participate in pollen development and polyester formation in the anther cuticle in maize remains unknown.

Although many male-sterile mutants in maize are available, very few of them have been functionally characterized [38]. *MS26* [39] and *MS45* [40], which have been functionally characterized, are required for pollen wall development. *MS26*, a homolog of *CYP704B1* in *Arabidopsis* and *CYP704B2* in rice, encodes a putative cytochrome P450 mono-oxygenase [41] that participates in the ω-hydroxylation of C16 and C18 fatty acids [26, 42]. *MS45*, which is expressed in the tapetal layer, encodes a putative strictosidine synthase involved in alkaloid biosynthesis [43, 44]. Maize *IPE1* (*IRREGULAR POLLEN EXINE1*), encoding a putative glc-methanol-choline oxidoreductase, participates in the oxidative pathway of C16/C18 ω-hydroxy fatty acids. IPE1, MS26, and MS45 might cooperatively mediate anther cuticle and pollen exine development [45]. The maize CYP703A2 subfamily member, APV1 (ABNORMAL POLLEN VACUOLATION1), also participates in the fatty acid hydroxylation pathway, which contributes to the biosynthesis of cutin monomers and sporopollenin precursors [46].

Here, we identified the maize male-sterile mutant *shrinking anther 1* (*sa1*), which displays abnormal tapetum and anther cuticle development. Allelism tests confirmed that *sa1* is allelic to the classical maize male sterile mutant *ms33*. However, the *MS33* gene has not yet been cloned. In the current study, we used map-based cloning to isolate the *MS33* gene and found that it encodes a putative GPAT. *MS33* was temporally expressed in the tapetum during anther development. The anthers of the *MS33* mutant had substantially reduced cutin and wax contents compared to wild type. Additionally, transcriptomic analysis revealed differentially expressed genes (DEGs) involved in wax, cutin, and fatty acid biosynthesis. Our findings suggest that MS33 may play an essential role in anther cuticle and pollen grain development in maize.

## Results

### Characterization of the male-sterile mutant *sa1*

**Fig. 1:**
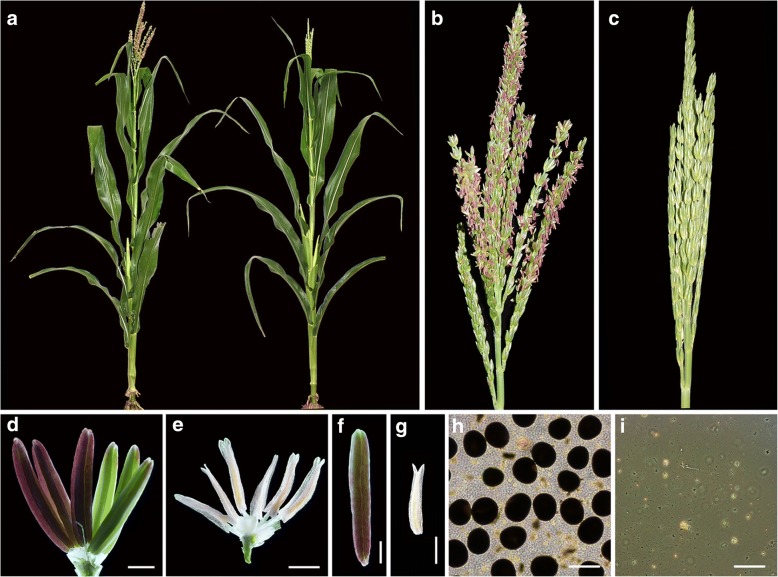
Phenotypic comparison of wild-type and *sa1* plants. **(a)** Wild-type plant (left) and *sa1* plant (right). **(b)** Wild-type inflorescence. **(c)** *sa1* inflorescence. **(d)** Wild-type flower. **(e)** *sa1* flower. **(f)** Wild-type anther. **(g)** *sa1* anther. **(h)** Viable pollen grains from a wild-type plant after I_2_-KI staining. **(i)** Nonviable pollen grains from an *sa1* plant after I_2_-KI staining. Bar =1 mm in (**d**), (**e**), (**f**), and (**g**), 50 μm in (**h**) and (**i**)

A male-sterile mutant, *shrinking anther 1* (*sa1*), was identified by screening a *MuDR* library [47]. This mutant exhibited normal vegetative development (Fig. 1a), whereas the anthers, which were not extruded from the spikelets, were small and wilted and failed to produce pollen grains (Fig. 1c, e, g, i). When *sa1* was pollinated by wild-type plants, seed production was normal and all F_1_ plants were fertile. The F_2_ plants showed a phenotype segregation ratio of 3:1 (fertile: sterile = 153:55, χ^2^ < χ^2^ (0.05, 1) = 3.84). These data suggest that female fertility is not affected in *sa1* and that its phenotype is due to a single recessive gene.

**Fig. 2:**
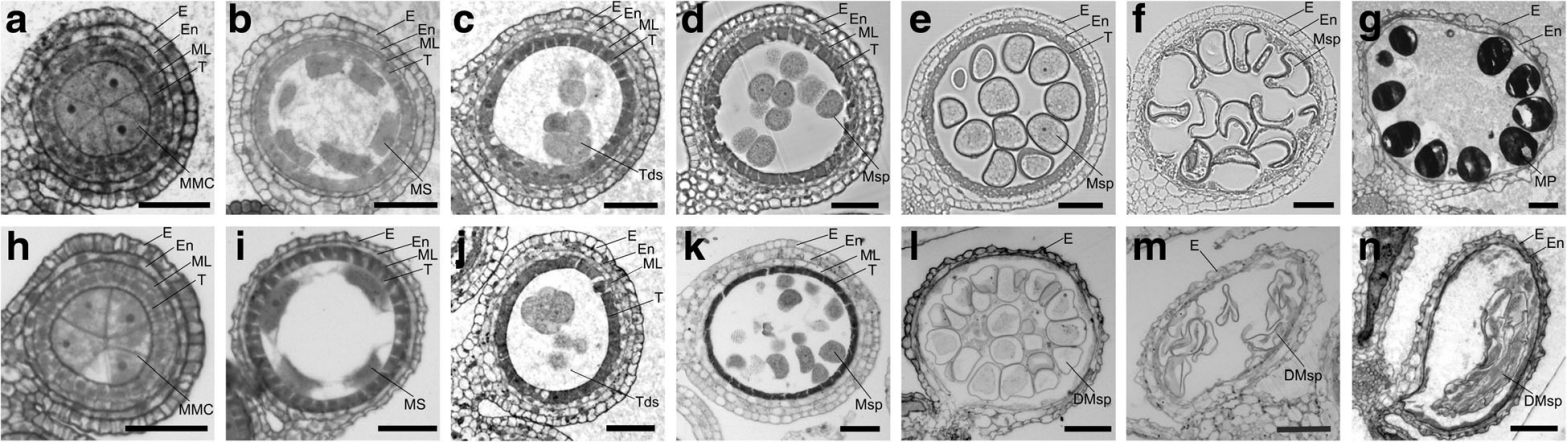
Transverse sections showing anther development in wild-type and *sa1* plants. Transverse sections of wild-type anthers are shown in a-g, and those of *sa1* anthers are shown in h-n. **(a)** and **(h)**, premeiosis stage. **(b)** and **(i)**, meiocyte stage. **(c)** and **(j)**, tetrad stage. **(d)** and **(k)**, early uninucleate stage. **(e)** and **(l)**, late uninucleate stage. **(f)** and **(m)**, binucleate stage. **(g)** and **(n)**, mature pollen grain stage DMsp, degenerated microspores; E, epidermis; En, endothecium; ML, middle layer; MMC, microspore mother cell; Mp, mature pollen; Ms., meiospores; Msp, microspore; T, tapetum; Tds, tetrads. Bars = 50 μm

To further examine the defects in *sa1*, we examined semi-thin transverse sections to compare the cytology of wild-type versus *sa1* anthers and found no difference in anatomical structure or meiotic events in the wild-type and *sa1* plants before the tetrad stage (Fig. 2a, b, c, h, i, j). The *sa1* anthers contained four somatic layers, and meiocytes were able to undergo normal meiosis II to form detectable tetrads (Fig. 2j).

At the early uninucleate stage, wild-type anthers had a thin middle layer, and the tapetum was degraded and appeared highly condensed (Fig. 2d). However, in *sa1*, the tapetal layer became thinner (Fig. 2k), pointing to more severe degradation of the tapetum. At the late uninucleate microspore stage, in wild-type anthers, the middle layer disappeared and the tapetal layer was degraded continuously but remained visible. Microspores, which contained full cytoplasm, gradually enlarged and became rounded (Fig. 2e). By contrast, in *sa1* anthers, the tapetum was almost completely degraded and barely any cellular material remained (Fig. 2l). This degeneration resembled that observed in *ipe1* [45]. The microspores of *sa1* appeared irregular. In wild-type plants, the tapetal layer exhibited a strip-like shape at the binucleate stage, and the microspores were still vacuolated (Fig. 2f). By contrast, in *sa1* anthers, the anther layers appeared collapsed and the microspores were defective (Fig. 2m). At the mature pollen grain stage, in wild-type anthers, many pollen grains were present in the anther locule (Fig. 2g), whereas in *sa1* anthers, no pollen grains were observed, and only some residual debris remained (Fig. 2n). These phenotypes suggest that SA1 may be involved in tapetum degradation and microspore development during anther development.

### SA1 is crucial for microspore and anther cuticle development

**Fig. 3:**
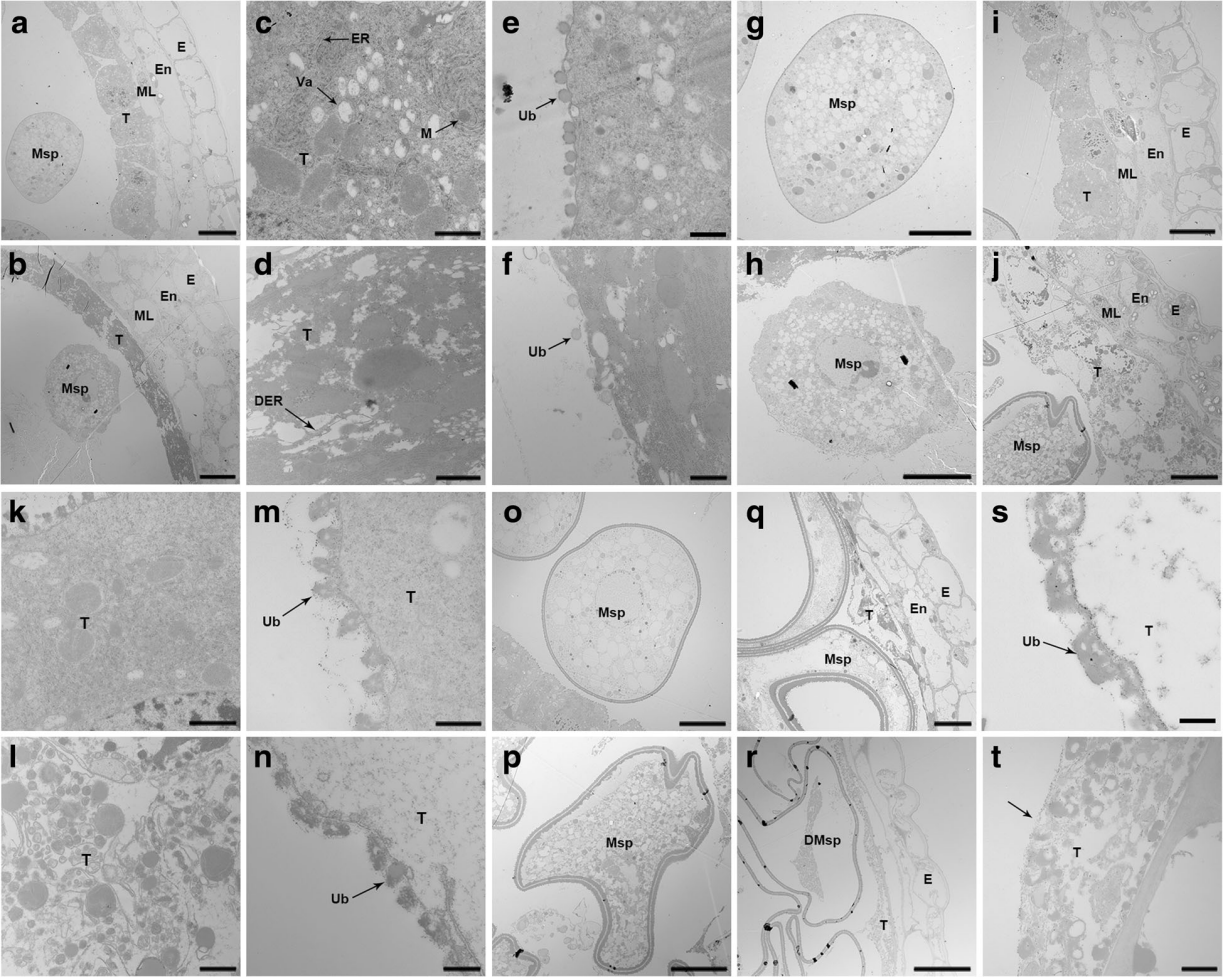
Transmission electron microscopy of anthers from wild-type and *sa1* plants. Early uninucleate stage anthers are shown in **(a)** to **(h)**: wild-type **(a)** and *sa1* anthers **(b)**; tapetal layers in wild type **(c)** and *sa1* **(d)**; Ubisch bodies in wild type **(e)** and *sa1* **(f)**; Microspores in wild type **(g)** and *sa1* **(h)**. Late uninucleate stage anthers are shown in **(i)** to **(p)**: wild-type **(i)** and *sa1* anthers **(j)**; tapetal layers in wild type **(k)** and *sa1* **(l)**; Ubisch bodies in wild type **(m)** and *sa1* **(n)**; microspores in wild type **(o)** and *sa1* **(p)**. Binucleate stage anthers are shown in **(q)** to **(t)**: wild-type **(q)** and *sa1* anthers **(r)**; Ubisch bodies in wild type **(s)** and *sa1* **(t)**. Bar = 10 μm in **(a)**, **(b)**, **(g)**, **(h)**, **(i)**, **(j)**, **(o)**, **(p)**, **(q),** and **(r)**; 1 μm in **(c)**, **(d)**, **(k)**, and **(l)**; 500 nm in **(e)**, **(f)**, **(m), (n), (s),** and **(t)**. DER, degenerated endoplasmic reticulum; DMsp, degenerated microspores; E, epidermis; En, endothecium; ER, endoplasmic reticulum; M, mitochondria; ML, middle layer; Msp, microspores; T, tapetal layer; Ub, Ubisch body; Va, vacuole

Transmission electron microscopy (TEM) revealed that at the early uninucleate microspore stage, wild-type tapetal cells contained numerous subcellular organelles, and Ubisch bodies were clearly visible in the inner surface of tapetum (Fig. 3a, c, e). However, in *sa1* plants, almost all subcellular organelles were degenerated, although Ubisch bodies were visible. Unlike the circular microspores of the wild type, *sa1* microspores were irregular in shape (Fig. 3b, d, f, g, h). At the late uninucleate microspore stage, the *sa1* tapetum was severely degraded, and no subcellular organelles or lipid bodies were observed (Fig. 3i, j, k, l). Ubisch bodies, which act as transporters for sporopollenin precursors between microspores and the tapetum, were slightly degraded in the *sa1* mutant (Fig. 3m, n). In addition, compared with the wild-type microspores, the *sa1* microspores were severely collapsed at this stage (Fig. 3o, p). At the binucleate microspore stage, the Ubisch bodies were degenerated in wild-type anthers and completely absent in *sa1* anthers (Fig. 3s, t). These results further indicate that SA1 may play crucial roles in microspore development and tapetal degradation.

**Fig. 4:**
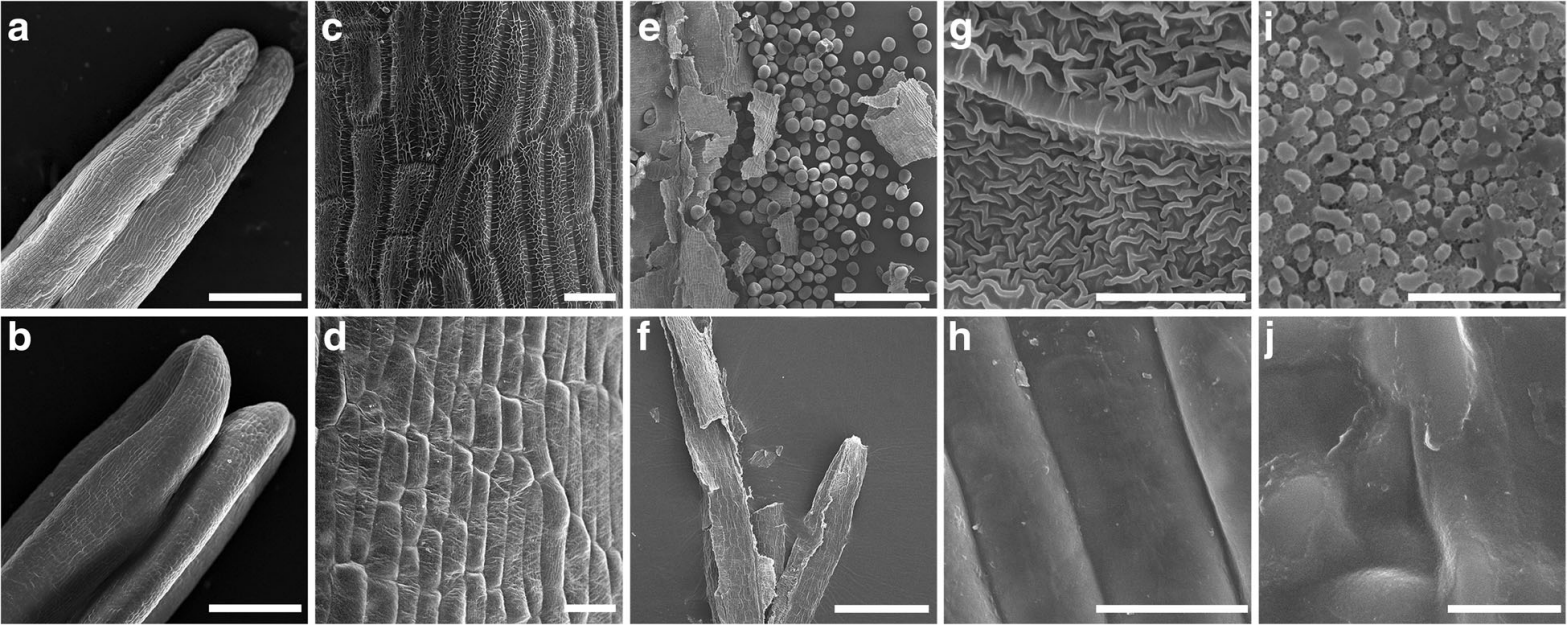
Scanning electron microscopy of wild-type and *sa1* anthers at binucleate and mature pollen grain stages**. (a)** and **(b)** The epidermal surfaces of a wild-type **(a)** and *sa1* anthers **(b)** at the binucleate stage. Bar = 250 μm. **(c)** and **(d)** Enlarged detailed view of the epidermal surfaces of anthers in wild type **(c)** and *sa1* **(d)** at the binucleate stage. Bar = 20 μm. **(e)** and **(f)** Many mature pollen grains are visible in wild type **(e),** but none are visible in *sa1* **(f)** at the mature pollen grain stage. Bar = 500 μm. **(g)** to **(j)** The outer **(g)** and inner **(i)** surfaces of wild-type anthers compared to the outer **(h)** and inner **(j)** surfaces of *sa1* anthers. The outer surface of the *sa1* anther is glossy **(h),** and no Ubisch bodies are visible on the inner anther surface **(j)**. Bar =10 μm in **(g)** and **(h)**, 5 μm in **(i)** and **(j)**

We then used scanning electron microscopy (SEM) to further investigate anther cuticle formation in *sa1* plants. At the late binucleate microspore stage, the cuticular ridges showed a stereoscopic knitting-like pattern in the wild-type plants (Fig. 4a, c). However, in the *sa1* mutant, the anther epidermis was smooth, with no obvious ridges; the anther epidermis had a slightly shrunken appearance (Fig. 4b, d). At the mature pollen grain stage, abundant pollen grains were observed in the wild type, whereas no pollen grains were detected in *sa1* anthers (Fig. 4e, f). In wild-type plants, reticulate cuticle covered the outermost surface and abundant Ubisch bodies were distributed on the outside of tapetal cells. By contrast, the outer surfaces of *sa1* anthers were glossy, and no Ubisch bodies were detected on the inner surface (Fig. 4g, h, i, j). These results suggest that anther cuticle development was disrupted in *sa1*.

### Map-based cloning of *SA1*

**Fig. 5:**
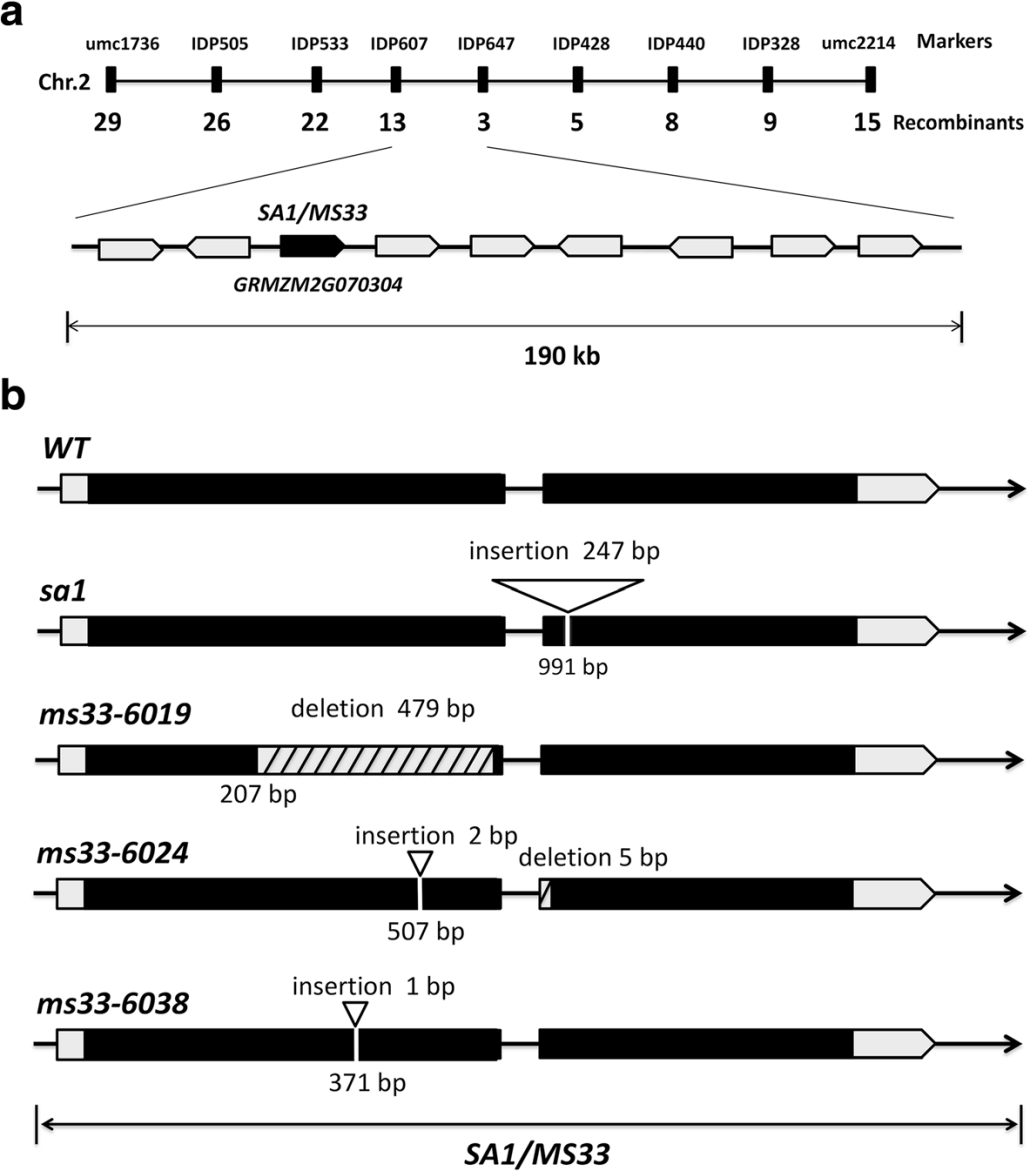
Map-based cloning and gene structure of *SA1/MS33.* **(a)** Fine mapping of the *SA1* gene on chromosome 2. Molecular markers and genes in the mapping region are indicated **(b)** Schematic representation of the structure of maize *SA1* in the wild type, *sa1*, *ms33–6019*, *ms33–6024*, and *ms33–6038*

We performed map-based cloning to isolate the *SA1* gene using 273 male-sterile plants from a segregating F_2_ population. *SA1* was initially mapped to a 1.84-Mb interval between two markers, umc1736 and umc2214, on chromosome 2 (Fig. 5a). Then, a number of InDel markers were developed for fine mapping using 2871 mutant individuals from the F_2_ population. The target region, including nine predicted open reading frames (ORFs), was narrowed down to a 190-kb interval between markers IDP607 and IDP647 (Fig. 5a). One of the nine ORFs, *GRMZM2G070304,* which spans 2161 bp and has two exons and one intron, encodes a putative GPAT with 525 amino acids. Sequence analysis of *sa1* genomic DNA revealed a 247-bp insertion in the second exon of *GRMZM2G070304* (Fig. 5b).

In a previous study, another recessive male-sterile mutant, *ms33*, was also mapped near this region, but the gene was not isolated [38]. The phenotype of *ms33* is very similar to that of *sa1* [48]. Therefore, we performed an allelism test to determine whether *ms33* is allelic to *sa1*. An *sa1* homozygote was pollinated by a fertile heterozygote (*+/ms33*) from the *ms33–6019* allelic line. The progeny exhibited a fertile: sterile segregation ratio of 1:1, suggesting that *ms33* is allelic to *sa1*. Sequencing analysis revealed that *ms33–6019* had a 479-bp deletion in the first exon of *GRMZM2G070304* (Fig. 5b).

We also sequenced *GRMZM2G070304* from two additional *ms33* allelic lines: *ms33–6024* and *ms33–6038*. In *ms33–6024*, two base pairs were inserted at the 507th base pair from the translational start site of this gene, and there was a 5 base-pair deletion at the beginning of the second intron. In *ms33–6038*, one base pair was inserted after the 371th base pair. Bioinformatic analysis predicted that these mutations in *ms33–6024* and *ms33–6038* might result in a frameshift and premature translational termination. These results suggest that the *sa1/ms33* phenotype is likely caused by the mutations in *GRMZM2G070304*.

### Phylogenetic analysis of MS33

GPATs have been characterized in bacteria, fungi, animals, and plants. These enzymes catalyze the transfer of an acyl group from acyl-CoA/ACP to the sn-1/2 position of glycerol-3-phosphate (G3P), i.e., the first step of de novo synthesis of membrane and storage lipids [49]. In *Arabidopsis*, 10 genes encoding GPAT enzymes have been annotated. GPAT1–GPAT8 clustered together in a single family based on sequence similarity analysis. This family has been found only in land plant species and shows sn-2 catalytic activity [8].

To identify the GPAT family members in maize and to explore the evolutionary role of MS33, we used the 10 *Arabidopsis* GPAT protein sequences as queries to search for their homologs in the maize and rice genomes by BLASTP against the Gramene database. Overall, 20 maize GPAT homologs including MS33 and 17 putative rice GPATs were identified. A neighbor-joining phylogenetic tree was then constructed (Additional file 1: Figure S1). The result shows that SA1/MS33 shares the highest similarity with rice Os11g45400. Both of them were clearly classified into the GPAT2/3 group.

### Expression pattern of *MS33*

**Fig. 6:**
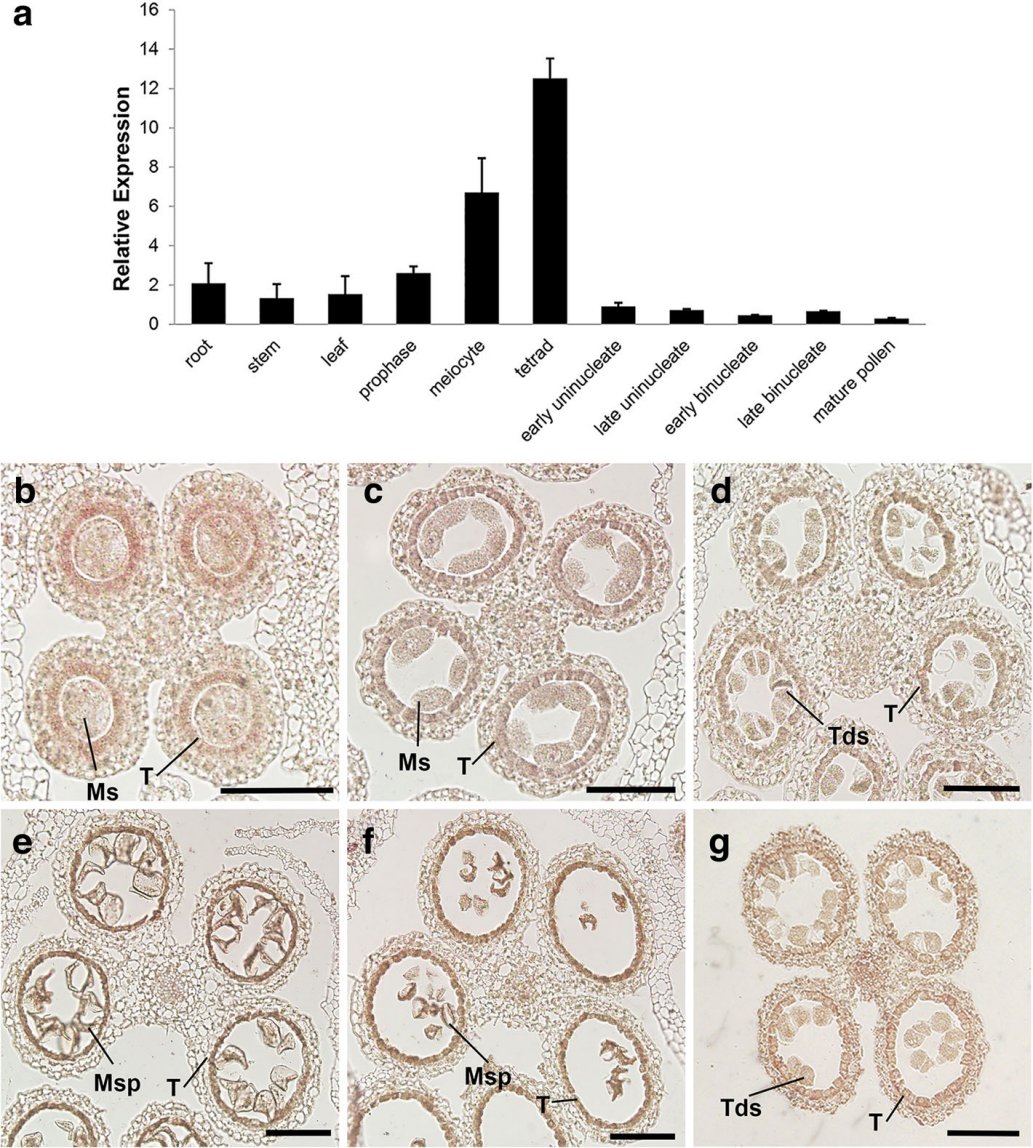
Expression pattern of *MS33****.*** **(a)** qRT-PCR analysis of *MS33* in different tissues and stages of pollen development. Data are presented as mean ± SE (*n* = 3). **(b**-**g)** In situ analysis of *MS33* expression in wild-type anthers. **(b)** to **(f)** *MS33* is highly expressed in tapetal cells of anthers at the premeiosis **(b)**, meiocyte **(c)**, tetrad **(d)**, uninucleate **(e),** and binucleate stages **(f)**. **(g)** anthers at tetrad stage hybridized with the *MS33* sense probe. Ms., meiospores; Msp, microspore; T, tapetum; Tds, tetrads. Bars = 100 μm

To further explore the function of MS33, we performed quantitative PCR (qPCR) analysis using total RNA extracted from various organs of wild-type plants. The qPCR assay detected *MS33* transcripts in all tissues examined, including anther, root, stem, and leaf tissue. Notably, *MS33* expression was dramatically stimulated in developing anthers, reaching a peak at the tetrad stage (Fig. 6a).

We then performed RNA in situ hybridization to investigate the spatiotemporal expression pattern of *MS33*. Strong *MS33* expression was detected in the tapetum from pre-meiosis to the binucleate stage (Fig. 6b-f). In the controls, no signal was detected when *MS33* sense probe was used (Fig. 6g). Similarly, previous studies have shown that *MS45* is specifically expressed in tapetal cells during early microspore development [38]. These expression patterns suggest that *MS33* might be involved in the synthesis of aliphatic materials required for anther cuticle and microspore development.

### The content and composition of cutin and wax are altered in *ms33* anthers

In *Arabidopsis*, GPATs participate in cutin and suberin biosynthesis. The levels of aliphatic polyester monomers are greatly reduced in leaves, stems, and flowers of GPAT mutants [8]. To investigate whether MS33 is involved in polyester monomer biosynthesis in maize anthers, we performed gas chromatography-mass spectrometry to measure the composition of aliphatic monomers in wild-type and *ms33* anthers.

**Fig. 7:**
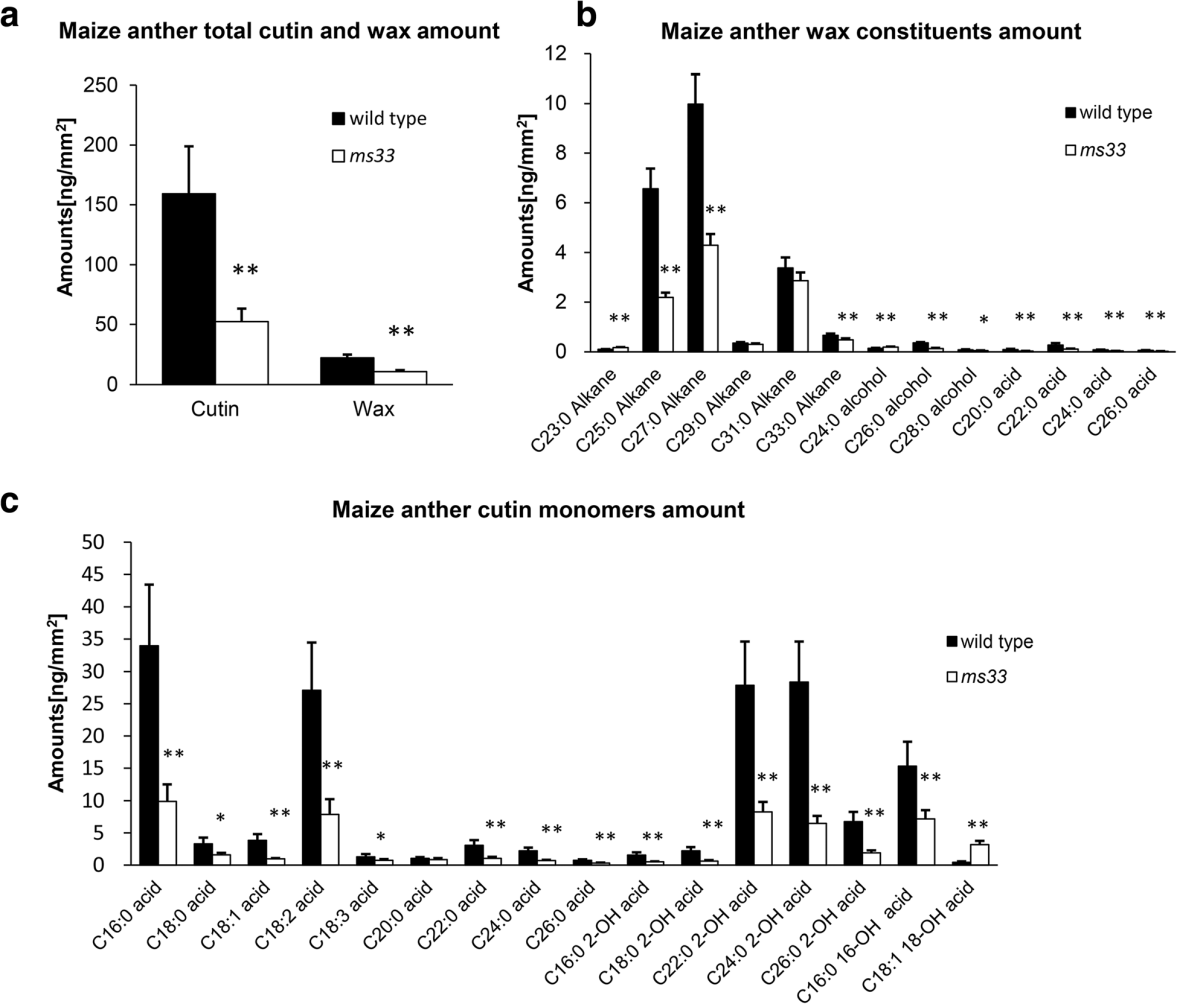
Analysis of anther wax and cutin monomers in the wild type and *ms33.* **(a)** Total cutin and wax level per unit of surface area in wild-type and *ms33* anthers. Error bars indicate SD (*n* = 5). *P < 0.05; **P < 0.01. **(b)** Wax constituent level per unit of surface area in wild-type and *ms33* anthers. Error bars indicate SD (*n* = 5). Compound names are abbreviated as follows: C23:0 Alkanes, tricosane; C25:0 Alkanes, pentacosane; C27:0 Alkanes, heptacosane; C29:0 Alkanes, nonacosane; C31:0 Alkanes, hentriacontane; C33:0 Alkanes, tritriacontane; C24:0 alcohol, 1-tetracosanol; C26:0 alcohol, 1-hexacosanol; C28:0 alcohol, octacosanol; C20:0 acid, eicosanoic acid; C22:0 acid, docosanoic acid; C24:0 acid, tetracosanoic acid; C26:0 acid, hexacosanoic acid. Error bars indicate SD (*n* = 5). *P < 0.05; **P < 0.01. **(c)** Cutin constituent level per unit of surface area in wild-type and *ms33* anthers. Error bars indicate SD (*n* = 5). Compound names are abbreviated as follows: C16:0 acid, palmitic acid; C18:0 acid, stearic acid; C18:1 acid, oleic acid; C18:2 acid, linoleic acid, C18:3 acid, linolenic acid; C20:0 acid, eicosanoic acid; C22:0 acid, docosanoic acid; C24:0 acid, tetracosanoic acid; C26:0 acid, hexacosanoic acid; C16:0 2-OH acid, 2-hydroxy-palmitic acid; C18:0 2-OH acid, 2-hydroxy-octadecanoic acid; C22:0 2-OH acid, 2-hydroxy-docosanoic acid; C24:0 2-OH acid, 2-hydroxy-tetracosanoic acid; C26:0 2-OH acid, 2-hydroxy-hexacosanoic acid; C16:0 16-OH acid, 16-hydroxy-hexadecanoic acid; C18:1 18-OH acid, 18-hydroxy-9-octadecenoic acid. Error bars indicate SD (*n* = 5). *P < 0.05; **P < 0.01

As shown in Fig. 7a, compared to wild-type anthers, total wax and cutin levels were significantly reduced in *ms33* anthers (by 51 and 67%, respectively). Among wax components, the amounts of the two predominate monomers in anthers, C25 and C27 alkanes, decreased by 67 and 57%, respectively (*P* < 0.01) in *ms33* compared to wild type (Fig. 7b, Additional file 2: Table S1). Among cutin components, C16:0 acid, C18:2 acid, C16:0 16-OH acid, C22:0 2-OH acid, and C24:0 2-OH acid are the major types of aliphatic cutin monomers in anthers. The levels of these monomers were substantially reduced (by 71, 71, 53, 70, and 77%, respectively) in *ms33* compared to wild type (Fig. 7c, Additional file 3: Table S2). These results suggest that MS33 may play an important role in the biosynthesis of wax and cutin monomers, which are essential for normal development of the anther cuticle and pollen grains.

We also compared the total fatty acid levels in wild-type versus *ms33* anthers. Total fatty acids with carbon lengths from C16 to C28 were present at 18.18 μg mg^− 1^ in *ms33* anthers but only 9.97 μg mg^− 1^ (*P* < 0.01) in wild-type anthers. The levels of the majority of fatty acids increased to various extents in *ms33* (Table 1). However, the octacosanoic acid (C28:0) level in wild-type anthers was 0.05 μg mg^− 1^, whereas none was detected in *ms33*. Similarly, eicosanoic acid (C20:0) levels in *ms33* decreased by approximately 20% (*P* < 0.01) compared to wild-type anthers. The differences in fatty acid monomer contents between wild type and *ms33* suggest that MS33 may play an important role in lipid biosynthesis pathways.

**Table 1:** Total fatty acids in wild-type and *ms33* anthers

Lipids	Wild type	*ms33*	Up
	*μg mg* ^*− 1*^ *dry weight*		
C16:1 acid	0.052 ± 0.009	0.094 ± 0.008	80.94%
C16:0 acid	4.128 ± 0.232	7.957 ± 0.116	92.74%
C18:2 acid	3.976 ± 0.114	7.786 ± 0.136	95.85%
C18:3 acid	0.466 ± 0.018	0.490 ± 0.012	5.22%
C18:1 acid	0.077 ± 0.005	0.134 ± 0.002	73.67%
C18:0 acid	0.531 ± 0.009	0.808 ± 0.029	52.17%
C20:0 acid	0.337 ± 0.015	0.269 ± 0.016	−20.22%
C22:0 acid	0.195 ± 0.014	0.345 ± 0.011	76.72%
C24:0 acid	0.109 ± 0.008	0.241 ± 0.005	120.29%
C26:0 acid	0.050 ± 0.005	0.054 ± 0.004	7.40%
C28:0 acid	0.053 ± 0.008	0	−100.00%
Total acids	9.973 ± 0.399	18.176 ± 0.257	82.26%

Fatty acid levels shown are means ± SD (*n* = 5)

### MS33 affects the expression of genes involved in wax and cutin biosynthesis

To further elucidate the molecular function of MS33, we conducted high-throughput transcriptome sequencing (RNA-seq) of wild-type and *ms33* anthers at the uninucleate stage, with three independent biological replicates. The percentages of mapped reads matching unique genomic positions were 71.05 and 70.17% in the wild type and *ms33*, respectively. Based on a false discovery rate (FDR) of < 0.05, 7953 of 39,604 genes were determined to be differentially expressed between the wild type and *ms33*, including 3335 downregulated and 4618 upregulated genes (Additional file 4: Figure S2a). According to Gene Ontology (GO) enrichment analysis, the downregulated genes are mainly involved in the categories carbohydrate metabolic process, lipid metabolic process, and fatty acid metabolic process, whereas the upregulated genes are primarily involved in the categories chemical stimulus, organic substance, and hormone stimulus (Additional file 4: Figure S2b). Notably, the DEGs include a number of genes involved in wax and cutin biosynthesis, such as *GLOSSY1*, *ZmFAR1*, and *ZmOCL1* (Additional file 4: Figure S2c) [50, 51], supporting the notion that MS33 functions in anther cuticle and pollen grain development.

## Discussion

### MS33 is required for anther cuticle and microspore development in maize

Previous studies on male-sterile mutants have shown that normal anther cuticle development is essential for male fertility [9]. The anther cuticle is primarily composed of two lipophilic biopolymers, cutin and wax [52]. Studies on partial depolymerization have demonstrated that cutin is mainly formed through the direct esterification of C16 and C18 fatty acids to glycerol or to each other [42]. Cuticular wax usually consists of aliphatic compounds with a chain length of at least 20 carbons, including alkanes, alcohols, very long chain fatty acids, and esters [34]. In rice and *Arabidopsis*, many enzymes and transcription factors involved in fatty acid biosynthesis also participate in cutin and wax synthesis during anther development. Mutations in these genes often lead to significant reductions in polyester monomer levels in anthers, particularly C16 and C18 monomers [9, 10].

In maize, *MS45*, *MS26*, *IPE1*, and *APV1* are required for anther cuticle development [45, 46]. Defects in these genes result in tapetum degeneration during early microspore development. Similarly, we found that *ms33* anthers had smooth surfaces and severe degradation of the tapetal layer during early microspore development compared with wild-type anthers. The anther cuticle is composed of wax and cuticular cutin, both of which are synthetized in the tapetum [9, 10]. Accordingly, our in situ hybridization showed that *MS33* transcripts were mainly found in the tapetum (Fig. 6b). Furthermore, mutations in *MS33* considerably altered the contents and composition of cutin and wax (Fig. 7). Therefore, we hypothesize that MS33 may be essential for anther cuticle development and that a mutation in *MS33* might alter aliphatic polyester biosynthesis in the tapetum.

To date, many male sterile mutants have been identified to be altered in polyester formation or synthesis of cutin and wax, and they are generally defective in both the anther cuticle and pollen exine, such as *ms2, cyp703a2,* and *gpat1 gpat6* double mutant in *Arabidopsis* [20, 36], *dpw*, *osgpat3, osnp1, cyp703a2,* and *cyp704b2* in rice [9, 10, 34, 37, 53], and *ms45*, *ms26*, *ipe1*, and *apv1* in maize [45, 46]. However, we found no significant structural differences in pollen exine between wild type and *ms33* at the uninucleate and binucleate stages, although *ms33* microspores had an irregular shape (Additional file 5: Figure S3). There are two possible explanations for these observations. On the one hand, perhaps *MS33* mediates pollen fertility independently because our RNA-seq analysis did not find differential expressions of *MS26*, *MS45*, *IPE1*, and *APV1* between wild type and *ms33* mutants (Additional file 6: Figure S4). On the other hand, perhaps the *ms33* pollen exine has been damaged in a way not detectable by TEM.

The *ms33* mutant displays defective anther cuticle development (Fig. 4). It has been proved that fatty acids are important precursors of cuticular cutin and wax [54]. Metabolism analysis revealed that *ms33* anthers had an increased level of the majority of fatty acids (Table 1). Therefore, we assumed that *ms33* controls cutin and wax metabolism by a complicated feedback regulation. The RNA-seq data are in accordance with this assumption, many genes are involved in fatty acids metabolism, such as fatty acids biosynthesis, fatty acids elongation and fatty acids oxygenation has been up or down regulated (Additional file 4: Figure S2c).

### The functions of GPAT family members are conserved and diverse in higher plants

GPAT-catalyzed de novo lipid biosynthesis has been extensively characterized in bacteria, fungi, and animals [55, 56]. In *Arabidopsis*, 10 GPATs have been identified, eight of which are specific to land plants and do not participate in membrane or storage lipid production [8]. Previous in vitro substrate specificity tests and phylogenetic analyses have shown that GPAT4/6/8 and GPAT5/7 are involved in cutin and suberin biosynthesis, respectively [35, 57, 58]. The *gpat4* and *gpat8* mutants exhibit reduced levels of C16 and C18 fatty acids and dicarboxylic acid cutin monomers in stems and leaves [49]. GPAT1/2/3 share a close evolutionary relationship and are expressed at relatively high levels in flowers. However, no obvious phenotype changes were detected in *gpat2* and *gpat3* mutants. AtGPAT2 and AtGPAT3 showed no activity on some fatty acid-CoA substrates [8, 59]. GPAT1 is active on the substrates of unsubstituted acyl-CoAs, including C16:0, C16:1, C18:0, C18:1, and C20:1 [59]. The *gpat1* mutant shows altered pollen coat structure and reduced fertility. GPAT6 is required for the incorporation of several C16 monomers into flower cutin [60]. The *gpat1 gpat6* double mutant exhibits abnormal pollen exine and tapetum structure, resulting in complete pollen abortion [36]. Therefore, GPAT family members may have diverse functions in the same species.

GPATs catalyze the initial step of glycerolipid biosynthesis by promoting the transfer of acyls from acyl-CoA or acyl-ACP to glycerol 3-phosphate at the *sn-2* hydroxyl to produce lysophosphatidic acid or monoacylglycerol, which are substrates for the synthesis of several important glycerolipid intermediates for cutin and wax production in plants [35, 61]. Unfortunately, the roles of GPATs in grasses are still unclear. Sixteen putative land-plant-specific GPATs have been identified in the rice genome [8], and 20 homologous genes were found in the maize genome in the current study (Additional file 1: Figure S1). However, to date, only one GPAT, OsGPAT3, has been characterized in rice [37]. In the current study, *MS33*, the first GPAT gene cloned from maize, was classified in the same clade with AtGPAT2/3 and OsGPAT3 based on phylogenetic analysis. The functions of *GPAT2/3* in *Arabidopsis* are still unknown [36]. Mutations in *OsGPAT3* may lead to abnormal tapetum and anther cuticle development, degenerated pollen grains, and reduced cutin and wax levels in rice, which is similar to our observations for *ms33*. In the current study, like *OsGPAT3* in rice, *MS33* transcripts were mainly detected in the tapetum during anther development in maize. The complete male sterility observed in *ms33* suggests that MS33 plays an essential role in microspore and anther cuticle development. Furthermore, the significant reduction in the levels of the major aliphatic monomers of cutin and wax in *ms33* anthers suggests that MS33 may be involved in anther cuticle biosynthesis. In addition, our transcriptome analysis revealed the differential expression of multiple genes involved in cutin and wax biosynthesis, including *FAE* and *KCS1* [62], *ZmOCL1* [51], *WSD1* [63], *CER5*/*WBC12* [64], *CER1* and *CER3* [65], *ZmWri1a*, *ZmWri1b*, and *WRINKLED1* [66–68] (Additional file 4: Figure S2). Therefore, MS33 in maize appears to share some common functions with GPAT family members in *Arabidopsis* and rice.

In higher plants, acyl-CoA is synthesized in the plastid and transported to the endoplasmic reticulum in cells in the tapetal layer [10]. We propose that in maize, MS33 may participate in polyester formation following fatty acid biosynthesis during early anther development. However, identification of the direct substrates and exact functions of MS33 will require further study.

## Conclusions

In this study, a male-sterile mutant *sa1*, which is allelic to the classic *ms33* mutant, displays defective anther cuticle development and premature microspore degradation. By a map-based cloning method, we isolated the *MS33* gene, which encodes a putative glycerol-3-phosphate acyltransferase (GPAT). The RNA in situ hybridization study showed that *MS33* was preferentially expressed in the tapetal layer cells during anther development. Using the gas chromatography-mass spectrometry (GC-MS), substantial reduction in wax and cutin were detected in *ms33* anthers. Accordingly, transcriptomic analysis demonstrated that many genes involved in wax and cutin biosynthesis were differentially expressed in *ms33* mutant. Taken together, our results suggest that *MS33* plays an important role in anther cuticle and microspore development by affecting lipid polyester biosynthesis. These findings provide insights into the function of glycerol-3-phosphate acyltransferase in the lipid polyester biosynthesis pathway and provide a potential male-sterile line for the utilization of heterosis in maize.

## Methods

### Plant materials

The *sa1* mutant in maize is a spontaneous mutant identified in the field, derived from breeding line HN17. The *ms33–6019*, *ms33–6024*, and *ms33–6038* mutants were obtained from the Maize Genetics Cooperation Stock Center. Two generations of the plants were cultivated per year; the summer generation was grown in an experimental field of China Agricultural University in Beijing, and the winter generation was grown in an experimental field in Sanya, Hainan. The BC_5_F_2_ population was created by backcrossing *sa1* to inbred line Z58 using linked markers. Morphological comparisons were performed within siblings from the same family in the BC_5_F_2_ population.

### Phenotypic analysis

The phenotypes of whole plants and reproductive organs were recorded using a Nikon E995 digital camera. Anthers from different developmental stages were collected based on anther length and microspore morphology. Phenotypic observations by semi-thin section, scanning electronic microscopy (SEM), and transmission electronic microscopy (TEM) were performed as described previously [10, 69].

### Map-based cloning

Two F_2_ mapping populations were obtained by crossing the *sa1* mutant with inbred line B73 or Z58, respectively, and positional cloning was carried out using both populations. Male-sterile plants were identified in the field, and genomic DNA was extracted from mature leaves of these plants using the cetyltrimethylammonium bromide method. Bulked segregant analysis [70] was performed using available SSR markers (www.maizegdb.org). Additional InDel markers were developed flanking the region identified by rough mapping [47]. The primer sequences are listed in Additional file 7: Table S3.

### Phylogenetic analysis

The full-length amino acid sequences of 10 *Arabidopsis* GPAT proteins were used as queries to identify their homologs in the maize and rice genomes via BLASTP (http://ensembl.gramene.org/Tools/Blast). In total, the sequences of 20 maize GPATs, 17 rice GPATs, and 10 *Arabidopsis* GPATs were used to construct a phylogenetic tree via bootstrap analysis with 1000 replications using MEGA 4.0 software [8].

### Real-time qRT-PCR

Tissue samples including leaves, roots, stems, and anthers of various lengths were separated and ground in liquid N_2._ Total RNA was extracted using an RNeasy Plant Mini Kit (Qiagen). The RNA was reverse transcribed using an M-MLV Reverse Transcription Kit (Invitrogen), and qPCR was performed using SYBR Green PCR Master Mix (Takara). Three biological replicates and three technical replicates were performed for each procedure. *ZmACTIN1* was used as the internal reference to normalize the expression data. Relative expression levels were calculated according to the 2^-ΔΔCT^ method [71]. The primer sequences are listed in Additional file 7: Table S3.

### In situ hybridization

Anthers at various developmental stage were separated according to length and developmental stage. Tissue fixation and in situ hybridization were performed as previously described [72]. The sequences of gene-specific primers are listed in Additional file 7: Table S3.

### Analysis of anther wax, cutin, and total fatty acids

Anthers at the uninucleate microspore stage from wild type and *ms33* were separated from stamens and immediately frozen in liquid N_2_. To determine the amount of each compound per unit surface area, the ratio of anther weight to surface area was calculated (Additional file 8: Figure S5). The surface area was calculated based on the length and width of the anther in microscopic images, assuming that maize anthers exhibit the standard cylindrical shape. Wax, cutin, and total fatty acid composition were analyzed as described previously [45]. Five biological replicates were performed per genotype.

### RNA-seq analysis

Anthers were collected from wild-type and *ms33* plants at the uninucleate microspore stage, with three biological replicates per genotype. Total RNA was isolated with TRIzol regent (Invitrogen). Sequencing libraries were constructed using a NEBNext Ultra RNA Library Prep Kit for Illumina (NEB, USA) and sequenced (paired-end, 150-bp reads) on a HiSeq 2500 sequencer. The raw data were filtered by removing reads containing adapters, reads containing poly-N, and low-quality reads to obtain at least six gigabases of clean data per sample. The clean data were aligned to the maize genome (AGPv3; MaizeSequence.org) using TopHat v2.0.12 [73] with default parameters. Gene expression levels were normalized by gene length and read numbers to calculate FPKM values (fragments per kilobase of transcript per million mapped reads). Significant DEGs were identified using the Cufflinks program [74]. The Singular Enrichment Analysis (SEA) tool in AgriGO [75] was utilized for GO enrichment analysis of the DEGs list, with default parameters. The DEGs were classified into functional categories defined by MapMan BINs (mapman.gabipd.org).

### Accession numbers

The maize GPAT genes referred to in this article can be found in the MaizeGDB/Gramene database under accession numbers GRMZM2G070304, GRMZM2G020320, GRMZM2G048561, GRMZM2G059637, GRMZM2G064590, GRMZM2G065203, GRMZM2G072298, GRMZM2G075295, GRMZM2G083195, GRMZM2G096010, GRMZM2G116243, GRMZM2G123987, GRMZM2G124042, GRMZM2G131378, GRMZM2G147917, GRMZM2G156729, GRMZM2G159890, GRMZM2G165681, GRMZM2G166176, and GRMZM2G177150.

The rice GPAT genes referred to in this article can be found in the Gramene database under accession numbers Os12g37600, Os11g45400, Os10g41070, Os10g27330, Os08g03700, Os05g38350, Os05g37600, Os05g20100, Os03g61720, Os03g52570, Os02g02340, Os01g63580, Os01g44069, Os01g22560, Os01g19390, Os01g14900, and Os10g42720.

*Arabidopsis* GPAT sequences can be found in the TAIR data libraries under the following accession numbers: AtATS1 (AT1G32200), AtGPAT1 (AT1G06520), AtGPAT2 (AT1G02390), AtGPAT3 (AT4G01950), AtGPAT4 (AT1G01610), AtGPAT5 (AT3G11430), AtGPAT6 (AT2G38110), AtGPAT7 (AT5G06090), AtGPAT8 (AT4G00400), and AtGPAT9 (AT5G60620).

## Additional files

Additional file 1: **Figure S1.** Phylogenetic analysis of MS33 and related homologs. MEGA 4.0 was used to construct the phylogenetic tree based on the neighbor-joining method. 10 *Arabidopsis* GPAT proteins, 17 rice GPAT proteins, and 20 homologs of *MS33* in maize were used for analysis and formed distinct clades. (TIF 796 kb)

Additional file 2: **Table S1.** Detailed wax compositions in wild-type and *ms33* anthers. (DOCX 15 kb)

Additional file 3: **Table S2.** Detailed cutin compositions in wild-type and *ms33* anthers. (DOCX 15 kb)

Additional file 4: **Figure S2.** Heat map representation of the differences in gene expression between the wild type and *ms33*. (a) Volcano plot of significant DEGs. X-axis: Log_2_ of the fold change in *ms33*/wild type, Y-axis: -log_10_ of the adjusted *P*-value. Red and green dots represent significantly up- and down-regulated genes, respectively (FDR < 0.01). Blue dots are genes with no significant change in expression. (b) GO functional categories of genes up- and downregulated in the indicated comparisons. The color of each cell indicates -log_10_ (*P*-values) of GO enrichment according to the scale shown. (c) Functional annotation and description of selected DEGs involved in anther cuticle development. For each gene, the FPKM value was normalized by the highest FPKM value of the gene across two samples. (TIF 10741 kb)

Additional file 5: **Figure S3.** Transmission electron microscopy of pollen exine in the wild type and *ms33*. (a) and (b), early uninucleate stage; (c) and (d), late uninucleate stage; (e) and (f), binucleate stage. Ex, exine. Bar = 500 nm in (a), (b), and (c), 1 μm in (d), (e) and (f). (TIF 1738 kb)

Additional file 6: **Figure S4.** *MS26, MS45, IPE1*, and *APV1* transcript levels in the wild type and *ms33*. Error bars indicate SD (*n* = 3). (TIF 9081 kb)

Additional file 7: **Table S3.** List of primers used in this study. (DOCX 15 kb)

Additional file 8: **Figure S5.** Weight/surface area ratios of wild type and *ms33* anthers. (TIF 3576 kb)

## Abbreviations

bp: base pair
InDel: Insertion/Deletion
qRT-PCR: quantitative real-time polymerase chain reaction
SRA: Sequence read archive
SSR: Simple sequence repeat
TAIR: The Arabidopsis Information Resource
