# Supplementary figures and images for: Maize male sterile 33 encodes a putative glycerol-3-phosphate acyltransferase that mediates anther cuticle formation and microspore development
Source: BMC Plant Biol. 2018 Dec 3;18:318. doi: 10.1186/s12870-018-1543-7 (PMC6276174; doi:10.1186/s12870-018-1543-7)

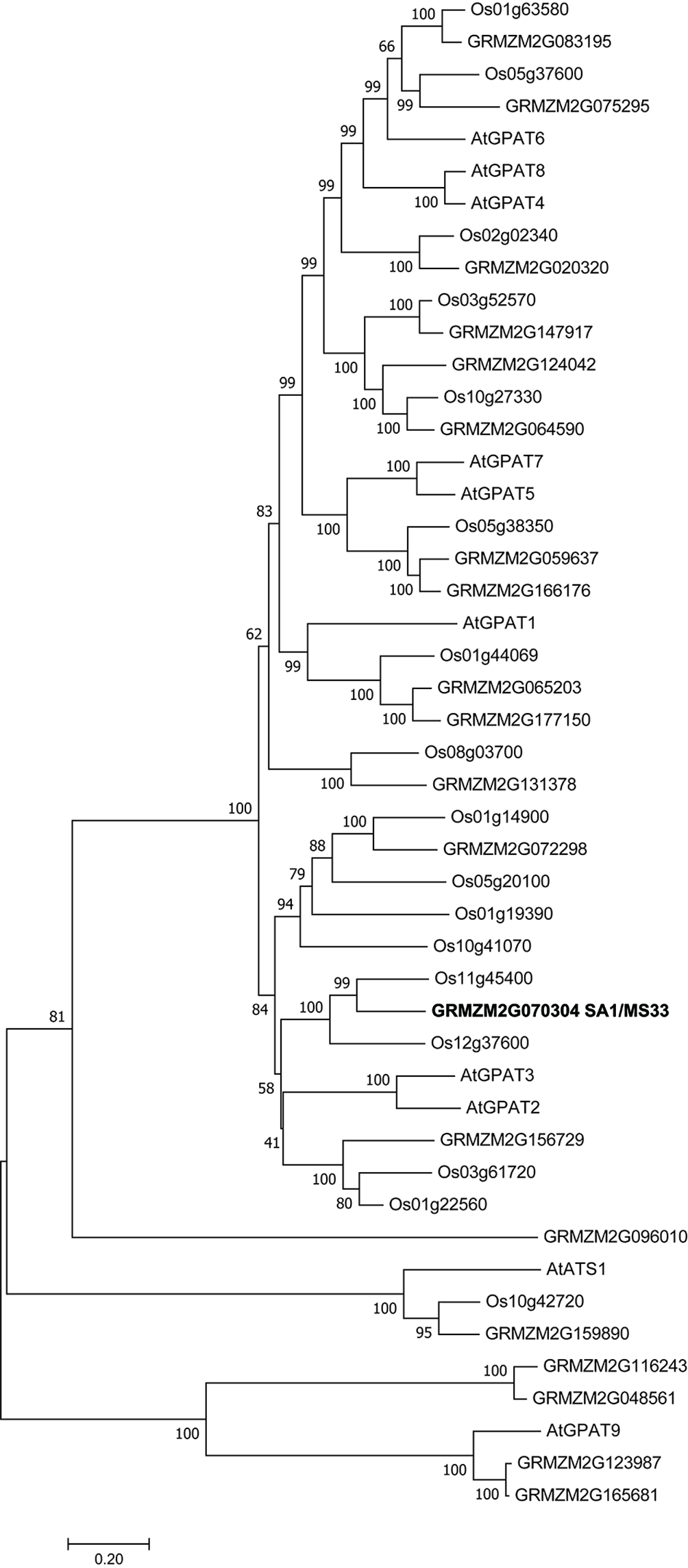

Supplement: Supplementary file 1 — Figure S1. Phylogenetic analysis of MS33 and related homologs. MEGA 4.0 was used to construct the phylogenetic tree based on the neighbor-joining method. 10 Arabidopsis GPAT proteins, 17 rice GPAT proteins, and 20 homologs of MS33 in maize were used for analysis and formed distinct clades. (TIF 796 kb) [file 12870_2018_1543_MOESM1_ESM.tif]

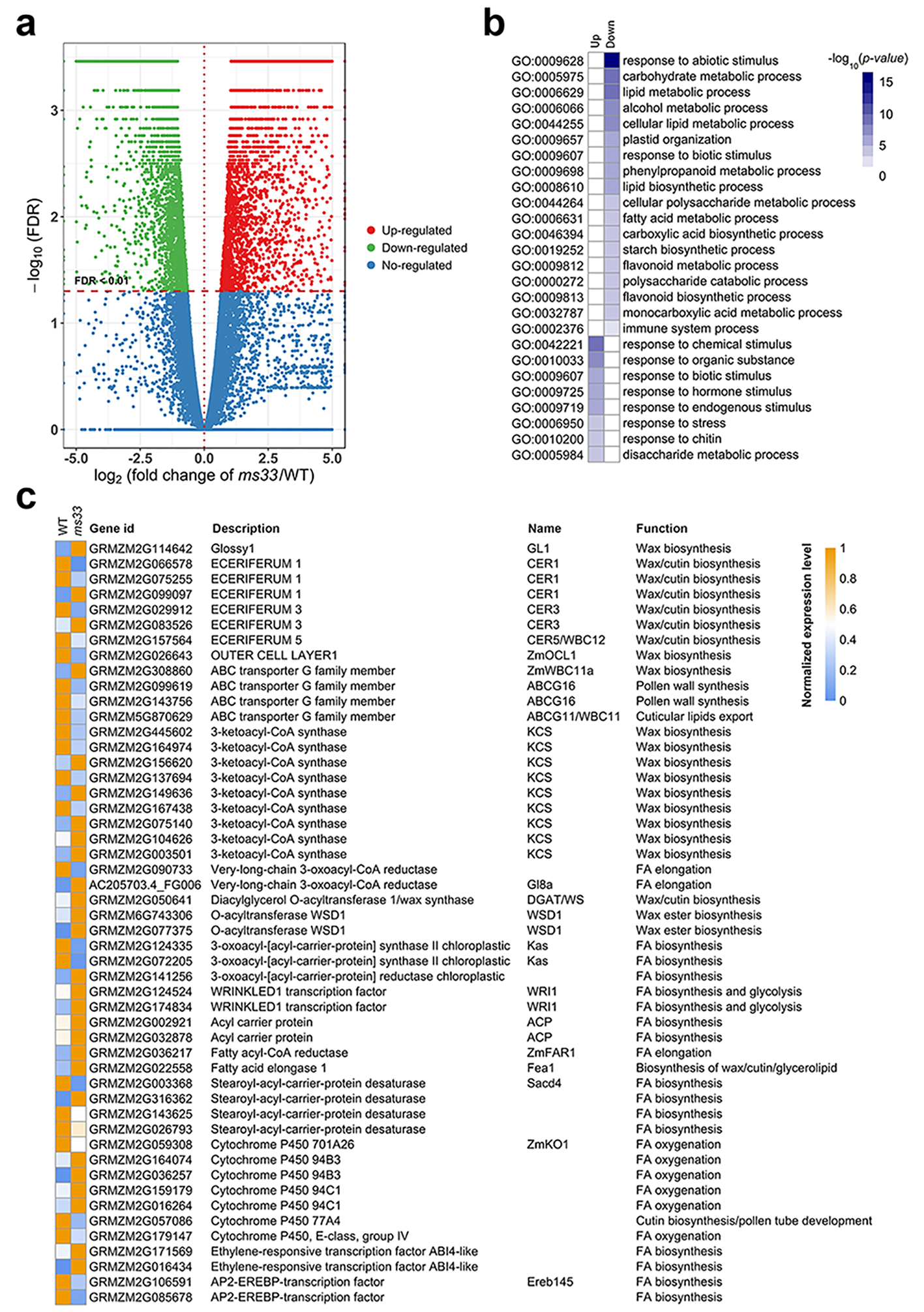

Supplement: Supplementary file 4 — Figure S2. Heat map representation of the differences in gene expression between the wild type and ms33. (a) Volcano plot of significant DEGs. X-axis: Log2 of the fold change in ms33/wild type, Y-axis: -log10 of the adjusted P-value. Red and green dots represent significantly up- and down-regulated genes, respectively (FDR < 0.01). Blue dots are genes with no significant change in expression. (b) GO functional categories of genes up- and downregulated in the indicated comparisons. The color of each cell indicates -log10 (P-values) of GO enrichment according to the scale shown. (c) Functional annotation and description of selected DEGs involved in anther cuticle development. For each gene, the FPKM value was normalized by the highest FPKM value of the gene across two samples. (TIF 10741 kb) [file 12870_2018_1543_MOESM4_ESM.tif]

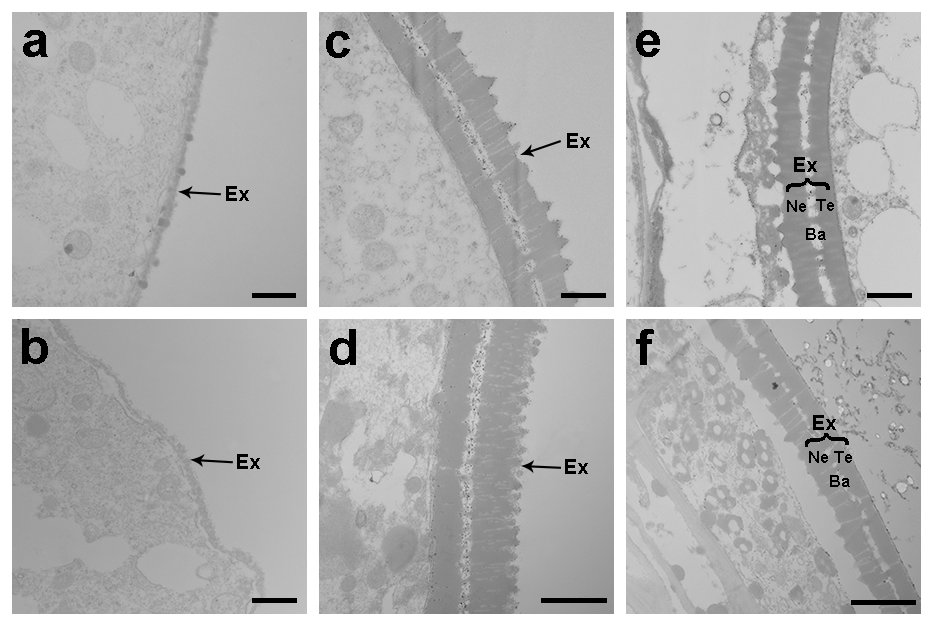

Supplement: Supplementary file 5 — Figure S3. Transmission electron microscopy of pollen exine in the wild type and ms33. (a) and (b), early uninucleate stage; (c) and (d), late uninucleate stage; (e) and (f), binucleate stage. Ex, exine. Bar = 500 nm in (a), (b), and (c), 1 μm in (d), (e) and (f). (TIF 1738 kb) [file 12870_2018_1543_MOESM5_ESM.tif]

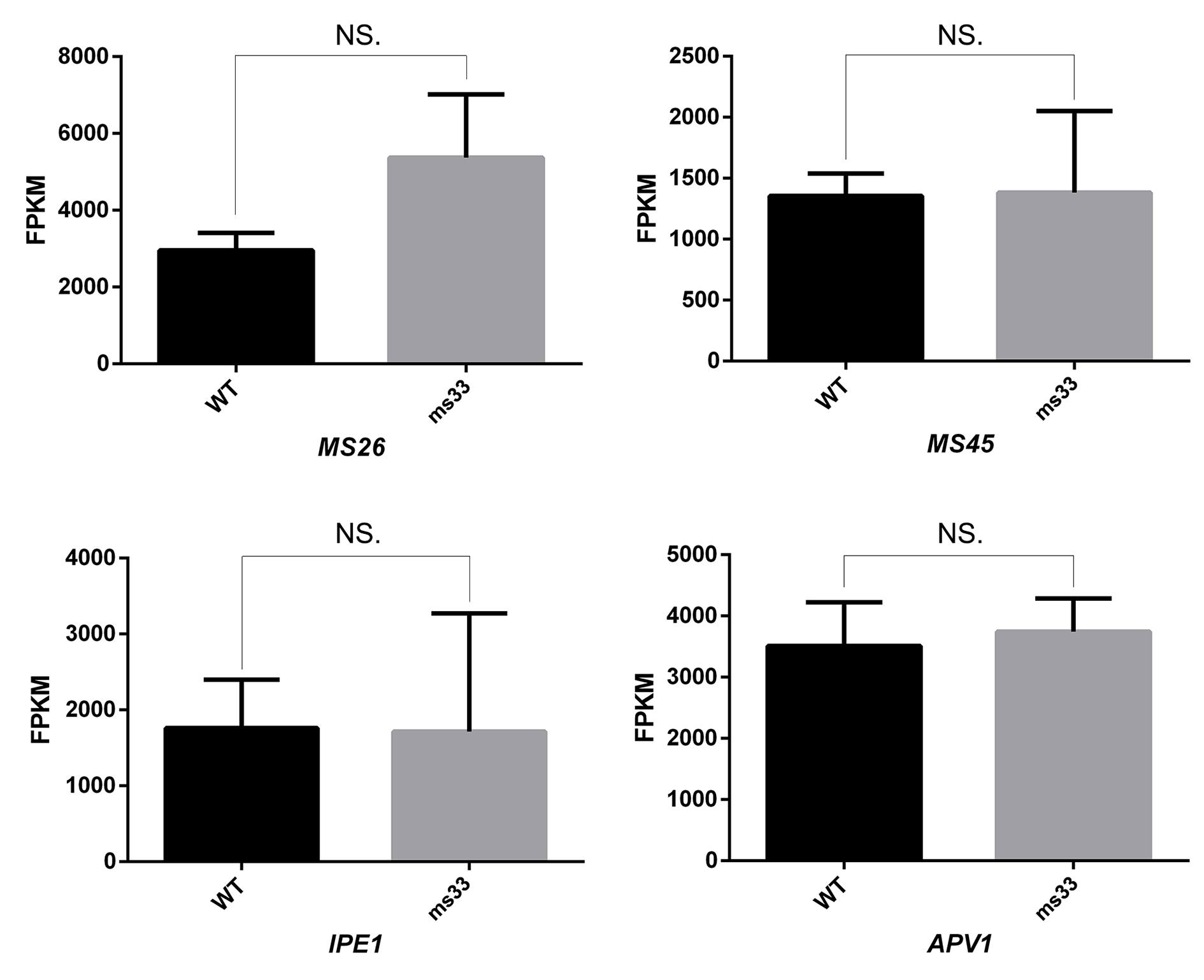

Supplement: Supplementary file 6 — Figure S4. MS26, MS45, IPE1, and APV1 transcript levels in the wild type and ms33. Error bars indicate SD (n = 3). (TIF 9081 kb) [file 12870_2018_1543_MOESM6_ESM.tif]
