# Supplementary material for: Maize male sterile 33 encodes a putative glycerol-3-phosphate acyltransferase that mediates anther cuticle formation and microspore development
Source: BMC Plant Biol. 2018 Dec 3;18:318. doi: 10.1186/s12870-018-1543-7 (PMC6276174; doi:10.1186/s12870-018-1543-7)
Supplement: Supplementary file 2 — Table S1. Detailed wax compositions in wild-type and ms33 anthers. (DOCX 15 kb) [file 12870_2018_1543_MOESM2_ESM.docx]

**Table S1. Detailed wax compositions in wild-type and *ms33* anthers**

| **Constituent** | **Wild type** | | ***ms33*** | |
| --- | --- | --- | --- | --- |
|  | **Mean (ng mm^-2^)** | **SD (ng mm^-2^)** | **Mean (ng mm^-2^)** | **SD (ng mm^-2^)** |
| C23:0 Alkane | 0.093 | 0.012 | 0.170 | 0.016 |
| C25:0 Alkane | 6.564 | 0.810 | 2.189 | 0.199 |
| C27:0 Alkane | 9.979 | 1.197 | 4.302 | 0.444 |
| C29:0 Alkane | 0.350 | 0.036 | 0.303 | 0.035 |
| C31:0 Alkane | 3.380 | 0.424 | 2.870 | 0.332 |
| C33:0 Alkane | 0.660 | 0.067 | 0.477 | 0.062 |
| C24:0 alcohol | 0.135 | 0.021 | 0.189 | 0.020 |
| C26:0 alcohol | 0.357 | 0.030 | 0.130 | 0.023 |
| C28:0 alcohol | 0.079 | 0.020 | 0.047 | 0.011 |
| C20:0 acid | 0.088 | 0.026 | 0.029 | 0.009 |
| C22:0 acid | 0.269 | 0.080 | 0.109 | 0.019 |
| C24:0 acid | 0.076 | 0.009 | 0.034 | 0.006 |
| C26:0 acid | 0.054 | 0.011 | 0.021 | 0.007 |

Data are presented as means ± SD (*n*=5).
