# Supplementary material for: Maize male sterile 33 encodes a putative glycerol-3-phosphate acyltransferase that mediates anther cuticle formation and microspore development
Source: BMC Plant Biol. 2018 Dec 3;18:318. doi: 10.1186/s12870-018-1543-7 (PMC6276174; doi:10.1186/s12870-018-1543-7)
Supplement: Supplementary file 3 — Table S2. Detailed cutin compositions in wild-type and ms33 anthers. (DOCX 15 kb) [file 12870_2018_1543_MOESM3_ESM.docx]

**Table S2. Detailed cutin compositions in wild-type and *ms33* anthers**

| **Constituent** | **Wild type** | | | | | ***ms33*** | | |
| --- | --- | --- | --- | --- | --- | --- | --- | --- |
|  | **Mean (ng mm^-2^)** | | **SD (ng mm^-2^)** | | **Mean (ng mm^-2^)** | | | **SD (ng mm^-2^)** |
| C16:0 acid | 33.961 | 9.462 | | 9.889 | | | 2.614 | |
| C18:2 acid | 27.082 | 7.353 | | 7.868 | | | 2.362 | |
| C18:3 acid | 1.311 | 0.397 | | 0.767 | | | 0.180 | |
| C18:1 acid | 3.837 | 0.994 | | 0.967 | | | 0.115 | |
| C18:0 acid | 3.313 | 0.944 | | 1.607 | | | 0.291 | |
| C20:0 acid | 1.045 | 0.225 | | 0.873 | | | 0.237 | |
| C22:0 acid | 3.086 | 0.784 | | 1.066 | | | 0.232 | |
| C24:0 acid | 2.212 | 0.531 | | 0.700 | | | 0.136 | |
| C26:0 acid | 0.743 | 0.149 | | 0.336 | | | 0.078 | |
| C18:0 2-OH acid | 2.221 | 0.585 | | 0.621 | | | 0.166 | |
| C16:0 2-OH acid | 1.560 | 0.429 | | 0.514 | | | 0.098 | |
| C22:0 2-OH acid | 27.867 | 6.716 | | 8.282 | | | 1.513 | |
| C24:0 2-OH acid | 28.358 | 6.258 | | 6.494 | | | 1.153 | |
| C26:0 2-OH acid | 6.753 | 1.497 | | 1.916 | | | 0.368 | |
| C18:1 18-OH acid | 0.452 | 0.131 | | 3.193 | | | 0.595 | |
| C16:0 16-OH acid | 15.314 | 3.792 | | 7.181 | | | 1.363 | |

Data are presented as means ± SD (*n*=5).
