# Supplementary material for: Maize male sterile 33 encodes a putative glycerol-3-phosphate acyltransferase that mediates anther cuticle formation and microspore development
Source: BMC Plant Biol. 2018 Dec 3;18:318. doi: 10.1186/s12870-018-1543-7 (PMC6276174; doi:10.1186/s12870-018-1543-7)
Supplement: Supplementary file 7 — Table S3. List of primers used in this study. (DOCX 15 kb) [file 12870_2018_1543_MOESM7_ESM.docx]

**Table S3. List of primers used in this study**

| **Primer name** | **Sequence (5'-3')** | **Used for** |
| --- | --- | --- |
| umc1736_F | CCATCCACCACTAGAAAGAGAGGA | Mapping |
| umc1736_R | TTAATCGATCGAGAGGTGCTTTTC | Mapping |
| ID_505_F | AGTGCTAATGCTTTTCATATCCGT | Mapping |
| ID_505_R | AGGTACCAGAAACATTTGGTCACG | Mapping |
| ID_533_F | AGGACATTAGCGGCAACCCTA | Mapping |
| ID_533_R | TGCCTTGACCTTGGAGACGAT | Mapping |
| ID_607_F | GTACGAACAGAAAACGGACCAC | Mapping |
| ID_607_R | CGCTCATCCATACCGTTAGCAG | Mapping |
| ID_647_F | CCCCACAATAAATGCAGTTCTCT | Mapping |
| ID_647_R | CCTGGATACAACATAGTGCCAT | Mapping |
| ID_428_F | ATATAGATTCCTTTAGTTCAATTCAG | Mapping |
| ID_428_R | GCTCGCTGCCAGCTTGTG | Mapping |
| ID_440_F | TACCATCCGCTTCGACTAC | Mapping |
| ID_440_R | CCACCTGCAGCAATAAACCATA | Mapping |
| ID_328_F | CCAGGAACCAGCACTAGGAA | Mapping |
| ID_328_R | TAGCTGCAGTGAACATGCCTT | Mapping |
| umc2214_F | ACCCCCTGATTCTCTCTTACGTTT | Mapping |
| umc2214_R | CTGGATGAGGAGGAAGAATACGAG | Mapping |
| q_sh1_F | GTCTGCGTCACTGCCATGCC | qPCR |
| q_sh1_R | CGGAGCTCACCACGAAGATCT | qPCR |
| q_ZmActin1_F | GAGATGCCTGATGGTCAGGTCA | qPCR |
| q_ZmActin1_R | AGTTGTACGTGGCCTCATGGAC | qPCR |
| antisense_sh1_SP6 | GATTTAGGTGACACTATAGAATGCT | RNA_*in_situ* |
|  | ATGGCCAAGAAGAGGCTC |  |
| antisense_sh1_T7 | TGTAATACGACTCACTATAGGGACG | RNA_*in_situ* |
|  | TCCTCCATGAGCCACTTG |  |
| sense_sh1_SP6 | GATTTAGGTGACACTATAGAATGCT | RNA_*in_situ* |
|  | TTGGGCAGCACGGCGCGGC |  |
| sense_sh1_T7 | TGTAATACGACTCACTATAGGGTCC | RNA_*in_situ* |
|  | TCCTACTAGCGCACAAGA |  |
